# Wastewater from the Edible Oil Industry as a Potential Source of Lipase- and Surfactant-Producing Actinobacteria

**DOI:** 10.3390/microorganisms9091987

**Published:** 2021-09-18

**Authors:** Pamela Welz, Gustav Swanepoel, Shandré Weels, Marilize Le Roes-Hill

**Affiliations:** Applied Microbial and Health Biotechnology Institute, Cape Peninsula University of Technology, P.O. Box 1906, Bellville 7535, South Africa; WelzP@cput.ac.za (P.W.); swangustav@gmail.com (G.S.); shandrelavern@gmail.com (S.W.)

**Keywords:** actinobacteria, biosurfactants, edible oil, microbiota, lipase, wastewater

## Abstract

Wastewaters generated from various stages of edible oil production in a canola processing facility were collected with the aim of determining the presence of lipase-producing actinobacteria of potential industrial significance. The high chemical oxygen demand (COD) readings (up to 86,700 mg L^−1^ in some samples) indicated that the wastewater exhibited the nutritional potential to support bacterial growth. A novel approach was developed for the isolation of metagenomic DNA from the oil-rich wastewater samples. Microbiota analysis of the buffer tank and refinery condensate tank wastewater samples showed a dominance of *Cutibacterium acnes* subsp. *defendens*, followed by a limited number of other actinobacterial genera, indicating the presence of a highly specialized actinobacterial population. Cultured isolates with typical actinobacterial morphology were analyzed for their ability to produce lipases and biosurfactants. Two strains, designated as BT3 and BT4, exhibited the highest lipase production levels when grown in the presence of tributyrin and olive oil (1.39 U mg^−1^ crude protein and 0.8 U mg^−1^ crude protein, respectively) and were subsequently definitively identified by genome sequencing to be related to *Streptomyces albidoflavus*. Cultivation of the strains in media containing different types of oils did not markedly increase the level of enzyme production, with the exception of strain BT4 (1.0 U mg^−1^ crude protein in the presence of peanut oil). Genome sequencing of the two strains, BT3 and BT4, revealed the presence of a range of lipase and esterase genes that may be involved in the production of the enzymes detected in this study. The presence of gene clusters involved in the production of biosurfactants were also detected, notably moreso in strain BT3 than BT4.

## 1. Introduction

Over the past one and a half centuries, numerous publications have focused on the enzyme lipase (triacylglycerol-acyl-hydrolase; EC 3.1.1.3). This ubiquitous enzyme is produced by animals, plants, and microorganisms and catalyzes the hydrolysis of glycerol-esters (notably long-chain acylglycerols vs. short-chain acylglycerols hydrolyzed by esterases) for the release of diacylglycerols, monoacylglycerols, and free fatty acids [1,2,3]. It is due to their ability to catalyze these reactions and their high degree of stereospecificity that they have found application in a wide range of industries: cosmetics, pharmaceuticals, detergents, food, leather, textile, paper, and biodiesel [3]. It is often found that organisms with the ability to produce lipases also produce surface active compounds [4]. These amphipathic compounds show activity at interfaces by lowering the tension between them: surfactants reduce surface tension at the air–water interface, while emulsifiers reduce the surface tension between two immiscible liquids. Lipases are known to facilitate the action of surfactants, and it is expected that some microorganisms isolated from oil-contaminated environments would have the ability to produce both lipases and surfactants [1,5]. With the need for more ‘green’ processes to drive the global bioeconomy, as well as consumer pressure, there is a continued search for affordable and safe natural surfactants and lipases. Even though numerous publications describe lipases from actinobacterial strains, there remains a gap in our knowledge of their complete complement of lipases and related enzymes, their potential for application in various industries, and their potential to be engineered to the level of commercially available lipases.

It has recently been shown that lipolytic microorganisms are present in lipid-rich industrial wastewater (dairy [6], chocolate factory [7], fish-canning [8,9]), as well as in domestic effluent [10,11] and landfill leachate [12]. Furthermore, in samples from ten environments historically polluted by lipid-containing wastewater, [13] found the highest concentration of lipase-producing bacteria in soil contaminated by edible oil (EO) industry wastewater. These studies were limited to the investigation of fast-growing fungi and bacteria (mostly *Enterobacteriaceae* and *Bacillus* species), with slower-growing microorganisms, such as actinobacteria, being excluded. However, weak lipase activity has been demonstrated in actinobacteria isolated from a domestic wastewater treatment plant [14].

In light of these studies, and due to the competitive advantage and selective pressure afforded by the ongoing presence of EO as a major carbon substrate, it was hypothesized that EO wastewater may be a potential source of industrially important lipase-producing actinobacteria. Edible oil is produced in bulk from seeds and fruit by various extraction and/or refining processes tailored to the type of feedstock and the desired characteristics of the product. Some of the production processes generate wastewater streams, including condensates (from solvent extraction, distillation, and deodorization), general wash-water (from spills, equipment cleaning, and truck wash-bays), and acid wastewater from soap-splitting [15]. The combined effluent typically has a high organic content, which can range from recalcitrant to highly biodegradable [16,17]. The organics in fresh effluent are naturally dominated by residual edible oils, which are measured as fats, oils, and grease (FOG). The FOG content is site-specific and is dependent on the processes and practices at each factory. Concentrations as low as <600 mg/L [16] to as high as >200,000 mg/L [18] have been reported.

This study was conducted in order to support our hypothesis that EO wastewater may be an important reservoir for the isolation of lipolytic actinobacteria. To achieve this, the macronutrient content of different EO wastewater streams was determined. Thereafter, heterotrophic plate counts and selective isolations for actinobacteria were performed using the streams that were deemed to contain suitable substrates to support bacterial growth. Actinobacterial isolates were (i) identified; (ii) screened qualitatively for their ability to produce lipases, biosurfactants, and haemolysins; and (iii) screened quantitatively for lipase and specific lipase activities. The specific lipase activities of the two most promising isolates were then compared after growth in a variety of EO-containing liquid media. Furthermore, the genomes of these isolates were sequenced, and the sequences interrogated for the presence of genes encoding for lipases, related enzymes, and biosynthetic gene clusters that could potentially encode for biosurfactants.

## 2. Materials and Methods

### 2.1. Sample Collection and Storage

Grab samples of wastewater from a canola EO industry were taken and transferred into sterile polypropylene containers. Samples included condensate from the hexane extraction plant (HE), acid wastewater from soap-splitting in the refinery (AW), condensate from the refinery (RC), wastewater from the buffer tank where acid wastewater and general wastewater were blended and neutralized (BT), and the separator tank (ST), where solids were gravity-settled and removed. Based on preliminary characterization results, fresh samples were taken from the RC, BT, and ST for bacterial enumeration, and actinobacterial isolation, identification, and screening for lipase activity (2.2–2.6). On receipt in the laboratory, the samples were thoroughly homogenized, and aliquots were either processed or frozen until required.

### 2.2. Physicochemical Analyses

The chemical oxygen demand (COD), total phosphorus (P), and total nitrogen (N) in the wastewater were measured using Merck (Darmstadt, Germany) cell tests together with a Merck Spectroquant^®^ instrument, with requisite standards and controls, according to the manufacturers’ instructions (COD cell tests for low, medium, and high range samples cat. nos. 1.14895.0001, 1.1451.0001, and 1.14691.0001, respectively; P cell test cat. no. 1.1453.0001, N cell test cat. no. 1.14537.0001). Samples were processed within 24 h of receipt in the laboratory. The FOG concentrations in the wastewater were determined using the partition gravimetric standard method 5520 B [19]. The fat and fatty acid composition of the EO and EO wastewater (2.5.3) were determined in duplicate using an Agilent (Santa Clara, CA, USA) gas chromatography flame ionization detector (GC-FID) instrument with a HP88 (100 m × 0.25 mm × 0.2 µm) column at a flow rate of 0.8 mL min^−1^ and an injection volume and temperature of 1 µL (split 50:1) and 250 °C, respectively. Nitrogen was used as the carrier gas, and the oven was programmed with an initial 2 min hold at 50 °C, thereafter increasing at 5 °C per min to 250 °C, followed by a 15 min hold at 250 °C.

### 2.3. Culture-Dependent Enumeration of Heterotrophic Bacteria and Isolation of Actinobacteria

Duplicate serial dilutions of the RC, BT, and ST EO wastewater were prepared in physiological saline, and 0.1 mL of each dilution was spread aseptically onto a range of culture media in Petri dishes. Plates were incubated in an inverted position aerobically at 30 °C and examined after 60 h. The colony-forming units (CFU) were enumerated on the plates containing 25–250 colonies. The plates were re-incubated for a further 2 weeks and examined every 2–3 days for colonies that morphologically resembled actinobacteria. Such colonies were sub-cultured to obtain pure growth on the same medium and culture conditions from which they were originally isolated. All media were made up to 1 L in distilled water and sterilized by autoclaving at 121 °C for 25 min: Czapek agar (g L^−1^: 30 sucrose, 3 Na_2_NO_3_, 3 MgSO_4_·7H_2_O, 0.5 KCl, 0.01 FeSO_4_·7H_2_O, 1 K_2_HPO_4_, 15 agar, pH 7.2); tributyrin agar (TBA) (g L^−1^: 5 peptone, 3 yeast extract (YE), 1% (*v*/*v*) tributyrin, 12 agar, pH 7.5); nutrient agar (NA) (g L^−1^: 5 peptone, 3 YE, 5 NaCl, 15 agar); starch-glycerol-glucose agar (SGG) (g L^−1^: 10 glucose, 10 potato starch, 10 glycerol, 2.5 corn steep powder, 5 peptone, 2 YE, 3 CaCO_3_, 1 NaOH, pH 7.0); Tween degradation agar (TDA) (g L^−1^: 10 peptone, 5 NaCl, 0.1 CaCl_2_, 10 mL Tween 80, 10 agar, pH 7.4); olive oil Rhodamine agar (OOR) (g L^−1^: 8 peptone, 1 YE, 3 NaCl, 10 mL olive oil, 0.002 Rhodamine B, 20 agar); and YE-malt extract agar (YEME) (g L^−1^: 10 malt extract, 4 YE, 4 glucose, 20 agar, pH 7.3). Pure cultures were maintained on YEME agar plates as well as 20% (*v*/*v*) glycerol stocks at −20 and −80 °C.

### 2.4. Identification of Actinobacteria

Actinobacterial isolates were cultivated in 10 mL liquid YEME at 30 °C, shaking at 160 rpm for cell mass production. The purity of the cultures was confirmed by standard Gram staining. A modified bead-beating method based on the procedure described by [20] was used for the isolation of total genomic DNA. Briefly, cell mass was harvested by centrifugation (10,000 rpm for 5 min) and the pellet washed with 1 mL 100 mM sodium phosphate buffer (pH 8.0). The washed pellet was resuspended in 300 μL phosphate buffer and frozen at −20 °C, overnight. The cell suspension was allowed to thaw and was transferred to a sterile 2 mL screw-cap vial containing 0.5 g 0.1–0.3 mm glass beads (Sigma-Aldrich, St. Louis, MO, USA). SDS lysis buffer (300 μL; 100 mM NaCl, 0.5 M Tris-HCl pH 8, 10%, *w*/*v*, SDS) was added and the suspension gently mixed, followed by the addition of 300 μL of chloroform:isoamyl alcohol (24:1, *v*/*v*). The suspension was mixed and incubated at room temperature (23 ± 2 °C) for three hours. The samples were then vortexed for 2 min and centrifuged at 13,200 rpm for 5 min. The supernatant was transferred to a sterile microfuge tube and 360 μL of 7 M ammonium acetate was added. The samples were shaken by hand and centrifuged at 13,200 rpm for 5 min. The supernatant was transferred to a sterile microfuge tube and 0.54 volumes of isopropanol added. The samples were incubated at room temperature (23 ± 2 °C) overnight and then centrifuged at 13,200 rpm for 5 min and the supernatant removed. The pellet was washed with 1 mL 70% (*v*/*v*) ethanol, centrifuged as before, and the pellet was allowed to air dry (15–45 min). The pellet was resuspended in 100 μL PCR-grade water. DNA samples were used for 16S rRNA gene sequence amplification. PCR was performed using the universal primers F1 (5′-AGAGTTTGATCITGGCTCAG-3′) and R5 (5′-ACGGITACCTTGTTACGACTT-3′), as per [21]. Amplicons were purified using the MSB^®^ Spin PCRapace kit (Stratec Molecular, Berlin, Germany) and submitted to Inqaba Biotechnical laboratories (Pretoria, South Africa) for sequencing. Sequences were submitted to EzBiocloud (https://www.ezbiocloud.net, accessed on 28 March 2019) to determine the classification of the actinobacterial isolates as well as their relatedness to type strains [22].

### 2.5. Amplicon Sequencing

Total metagenomic DNA was extracted using a combination of the Machery-Nagel (Düren, Germany) NucleoSpin^®^ Food genomic DNA isolation kit and the NucleoSpin^®^ Genomic DNA from soil kit, with modifications. The wastewater samples were filtered through polycarbonate filters (142 mm diameter), and the filters were cut into small pieces and transferred to sterile 15 mL Greiner tubes. The beads of three NucleoSpin^®^ Bead Tubes Type A (0.6–0.8 mm ceramic beads) were added to the pieces of filter. The lysis buffer, SL1 (2.8 mL), from the Soil DNA isolation kit was added to the filter pieces and beads and incubated at room temperature for 5 min. The samples were vortexed horizontally at maximum speed for 5 min. The mixture was centrifuged for 10 min at 8000× *g* and the lysate transferred to a clean tube. Buffer SL3 (Soil DNA isolation kit; 600 μL) was added to the lysate, vortexed for 5 s, and incubated at 4 °C for 10 min. Step-by-step, 700 μL of the lysate mixture was applied to the NucleoSpin^®^ Inhibitor Removal Column and centrifuged for 1 min at 11,000× *g*. The flow-through was collected in clean 15 mL tubes and mixed with 1 mL Buffer SB, vortexed thoroughly, and 550 μL of the lysate was loaded onto NucleoSpin^®^ Food Columns and washed, as per the protocol. DNA was eluted twice with 50 μL pre-warmed (70 °C) Elution Buffer. The amount of DNA was quantified using a Jenway Genova (Bibby Scientific, Staffordshire, UK) Nanodrop spectrophotometer.

Amplicon sequencing was performed at Inqaba Biotechnical laboratories (Pretoria, South Africa). Partial 16S rRNA bacterial gene sequences were amplified using Actinobacteria-specific 16S rRNA gene primers (Com2xf: 5′-AAACTCAAAGGAATTGACGG-3′; Ac1186r: 5′-CTTCCTCCGAGTTGACCC-3′) [23]. PCR reactions were carried out in 25 µL volumes containing 12.5 µL Q5^®^ Hot-start high fidelity 2X Mastermix (New England Biolabs, Boston, MA, USA), 1.25 µM each primer, and approximately 200 ng metagenomic DNA. PCR was performed on all replicate extractions separately using an ABI 2720 Thermal cycler (SeqGen, Torrance, CA, USA). The program used are as follows: 2 min at 95 °C for denaturation; 30 cycles of 20 s denaturation at 95 °C, 30 s annealing at 55 °C and 30 s elongation at 72 °C; and a final elongation step of 10 min at 72 °C. The amplicon libraries were purified using the Agencourt Ampure^®^ XP bead kit (Beckman Coulter, Brea, CA, USA) and sequenced on an Illumina MiSeq^TM^ (San Diego, CA, USA) instrument. Data sets were submitted to the 16S Microbiome Pipeline of EzBioCloud (https://www.ezbiocloud.net/, accessed on 21 April 2021) for analysis. The reads were filtered to remove low quality amplicons, non-target amplicons, and chimeric amplicons. Database version PKSSU4.0 was used in the analysis of the data.

### 2.6. Genome Sequencing and Analysis

Actinobacterial strains with the highest level of lipase activity, strains BT3 and BT4, were selected for genome sequencing. Both strains were cultivated in 10 mL YEME liquid media for 5 days, 30 °C, 160 rpm. Cell mass was harvested and DNA isolated as according to Mandel and Marmur [24]. The purified DNA was submitted for genome sequencing (Ion Torrent S5 platform) to the Central Analytical Facility, University of Stellenbosch, South Africa. The BAM output files were converted to FASTQ format by using bedtools v2.27.1. The genome sequences were assembled using SPAdes version 3.14.1 [25], and the resultant fasta file was submitted to the Rapid Annotation using Subsystem Technology (RAST version 2) server for annotation [26]. The assembled genomes were also submitted to TrueBac^TM^ ID (EzBioCloud) [27] in order to definitively determine the identity of strains BT3 and BT4. Lipase sequences were extracted from the annotated genomes and analysed using BLASTp (https://blast.ncbi.nlm.nih.gov/, accessed on 2 July 2021) [28] as well as SignalP-5.0 (https://services.healthtech.dtu.dk/service.php?SignalP, accessed on 2 July 2021) [29].

Genomes were submitted to antiSMASH 5.0 [30] to determine the presence of potential biosynthetic gene clusters involved in biosurfactant production and to dbCAN2 [31] to determine whether the strains exhibit the potential to degrade lignocellulosic materials. GenBank assembly accession numbers have been assigned to both genomes—BT3: GCA_016734865.1 (BioSample SAMN14167118) and BT4: GCA_016734825.1 (BioSample SAMN14167119).

### 2.7. Lipase Activity and Surfactant Production

#### 2.7.1. Screening Tests for Surfactant Production

In order to determine the ability of the actinobacterial isolates to produce biosurfactants, various standard screening assays were performed. In the assays where culture supernatant was used, the actinobacterial isolates were cultured in YEME liquid media as well as YEME supplemented with 0.1% (*v*/*v*) canola oil for 5 days, 30 °C, 160 rpm.

Haemolytic activity: Actinobacterial isolates were stab-inoculated into blood agar plates (5% *v*/*v* horseblood). The plates were incubated at 30 °C for 7 days and examined for a clear zone around the colonies [32].

Drop collapse test: 7 μL of oil (mineral, canola, olive) was added to 96-well microtiter plates and allowed to equilibrate overnight. To each oil type, 10 μL of culture supernatant was added. Distilled water, 10% (*w*/*v*) sodium dodeclysulphate, and growth media were used as controls. Intact oil droplets represented negative results, while if the culture supernatant caused the drop to collapse, it was recorded as a positive result [33].

Oil spreading assay: the oil spreading assay was determined as described by [33] and [34]. Distilled water (50 mL) was added to Petri dishes and 100 μL of mineral oil was added to the surface. Culture supernatant (10 μL) was pipetted onto the mineral oil and the diameter of the clear zone on the oil surface was measured.

Emulsification test: 1 mL of culture supernatant was mixed with 1 mL of kerosene. The mixture was vortexed for 2 min and allowed to stand for 24 h. The percentage of emulsification was calculated from the % height of the emulsion to the total height of the solution [35]. YEME, YEME supplemented with 0.1% (*v*/*v*) canola oil, distilled water, and Triton X-100 were used as controls.

#### 2.7.2. Screening Tests for Lipase Production

Actinobacterial isolates were screened for lipase activity on TDA, TBA, OOR, and egg yolk agar (EYA) (g L^−1^: 10 peptone, 5 YE, 10 NaCl, 10 mL 10% (*w*/*v*) glucose, 100 mL 50% (*v*/*v*) egg yolk, 20 agar). For the EYA, the glucose was filter-sterilized, and the egg yolks were emulsified aseptically in sterile distilled water in a sterilized stainless-steel blender. Both the glucose and egg yolk emulsion were added to the rest of the ingredients after the media had cooled to <60 °C. Isolates were spot inoculated (approximately 3 cm × 0.5 cm) onto the surface of dry agar plates, two isolates per plate. Plates were incubated for 10 days and examined every two to three days for activity. A positive result was determined as a zone of clearing around the growth on the TDA and TBA, and a ‘mother-of-pearl’ iridescence around the growth on the EYA.

#### 2.7.3. Lipase Activity Assays

Strains were plated onto YEME agar and incubated for 5 days at 30 °C. A loopful of growth from each strain was inoculated into sterile 7 mL of tributyrin/edible oil (TEO) broth (g L^−1^: 5 peptone, 3 meat extract, 10 mL tributyrin, 10 mL EO, 0.5 mL Tween 80 made up to 1 L in distilled water) in McCartney bottles containing approximately 20 glass beads 3 mm in diameter. The suspensions were homogenized by vortexing, and 1 mL of each suspension was aseptically transferred to six 50 mL Erlenmeyer flasks containing 10 mL of sterile TEO broth. The flasks were shaken on an orbital shaker at 37 °C at 160 rpm, except for isolates RC2 and BT7, which did not grow at 37 °C, and were therefore incubated at 30 °C. For the initial assays, olive oil was used in the TEO broth. One set of cultures was incubated for 5 days, and one set for 10 days, after which the lipase activity was determined. The procedure was repeated for isolates BT3 and BT4 using a range of EOs (olive, canola, sunflower, peanut, avocado), and an incubation period of 5 days. All assays were conducted in triplicate. The lipase activity assay was performed on whole cell cultures and well as supernatant fluid according to the method described by Palacios et al. [36], with the following modifications. To allow complete dissolution of the substrate, 4-nitrophenyl palmitate (4-NP, Sigma-Aldrich) was composed of equal volumes of isopropanol and acetonitrile (instead of pure isopropanol). The assay mixture consisted of 3.0 mL buffer and 0.6 mL culture supernatant (instead of 3.5 mL and 0.1 mL, respectively), and the reaction time was increased from 5 min to 10 min. One unit (U) was defined as the amount of enzyme required to release 1 µmol of nitrophenol from 4-NP in 1 min. Crude protein concentrations were determined by adding 0.1 mL of culture supernatant to 0.9 mL Bradford’s reagent (Sigma-Aldrich) and determining the absorbance at 595 nm using a spectrophotometer. Standard graphs were prepared using albumin from bovine serum (Sigma-Aldrich cat no: B6916). Specific activity was defined as the number of units of enzyme per mg of crude protein.

### 2.8. Statistical Analyses

Statistical analyses (*t*-tests) were performed using Microsoft Excel.

## 3. Results

### 3.1. Qualitative Analysis of Edible Oil Wastewater Streams

In this study, wastewater samples from a number of locations in a canola oil factory were screened using COD as a proxy for the organic nutrient concentration (Table 1). As expected, the COD of HE wastewater was low, and on the basis of these results, it was excluded from further analysis. The AW was also excluded on the basis of a low FOG concentration as well as the highly acidic nature (pH < 2) of this effluent stream [17], which precluded microbial growth during initial screening (data not shown). Further studies were conducted on the RC, BT, and ST wastewaters only. These all contained high concentrations of FOG, therefore making them promising candidates for the isolation of lipolytic actinobacteria. The COD:N ratios of BT and ST were 21,625:1 and 6400:1, respectively, and the N concentration in RC was below the detection limit of the analytical method (Table 1). They were therefore considered to be N-deficient (‘Ideal’ COD:N:P ratios of 100:5:1 and 250:5:1, respectively, are required for efficient aerobic and anaerobic microbial processes in wastewater treatment plants [37,38]). Unlike N, P was not a limiting nutrient in BT and ST, because phosphoric acid was added for degumming and neutralising during the oil refining process.

### 3.2. Enumeration of Culturable Heterotrophic Bacteria

**Figure 1:**
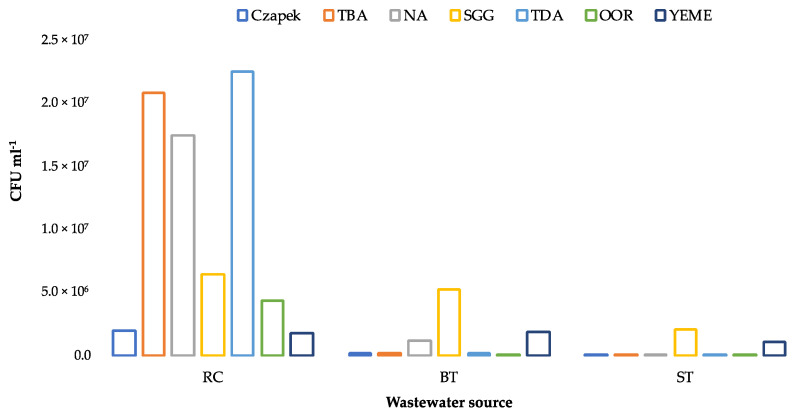
Bacterial counts obtained from selected wastewater samples on different media. TBA = tributyrin agar; NA = nutrient agar; SGG = starch, glycerol, glucose agar; TDA = tween degradation agar; OOR = olive oil Rhodamine agar; YEME = yeast extract malt extract agar; RC = refinery condensate; BT = buffer tank; ST = separator tank.

Despite the N limitation, growth was obtained from RC, BT, and ST wastewaters on all the selected culture media (Figure 1). Apart from YEME, significantly higher numbers (paired *t*-test, *p* < 0.05) of CFUs were obtained on cultures from RC than from BT or ST, and preferential growth patterns were observed on the RC culture plates (TDA > TBA > NA > SSG > OOR > YEME/Czapek). In terms of media composition, TDA and Czapek contained single carbon sources, while all the other media contained a more complex source (YE), either alone (NA), in combination with a simple sugar (YEME), oil (OOR), or multiple sources (SGG). There was a >2 log difference between the number of CFUs obtained on TDA vs. Czapek (Tween 80 vs. sucrose) and TBA vs.YEME (tributyrin + YE vs. glucose + YE), which strongly suggested that the tributyrin and Tween 80 were preferred over simple sugars as substrates for the heterotrophic bacteria in RC. This supported our hypothesis that the acclimation and selection of microbial communities capable of directly utilizing FOG takes place in EO wastewater.

The preferential growth patterns on the media from BT and ST differed from RC but were similar to one another. In each case, the highest number of CFUs were enumerated on SSG, followed by YEME (Figure 1).

### 3.3. Identification of Actinobacteria and Screening for Lipase and Biosurfactant Activity

All colonies morphologically resembling actinobacteria from the various isolation media were sub-cultured onto appropriate media to obtain pure growth and identified by sequencing of the 16S rRNA gene (Table 2). Actinobacteria were only isolated from the RC and BT effluent streams. A total of eleven strains were cultured, identified, and screened for their ability to produce biosurfactants and lipase (Table 2). Four different genera were represented amongst the strains: *Arthrobacter*, *Gordonia*, *Micromonospora*, and *Streptomyces*. The 16S rRNA gene sequences are available from GenBank under the following accession numbers: BT1 (MZ889014), RC1 (MZ889015), BT2 (MZ889016), BT3 (MZ889017), BT4 (MZ889018), RC2 (MZ889019), BT5 (MZ889020), BT6 (MZ889021), BT7 (MZ889022), BT8 (MZ889023), and BT9 (MZ889024).

**Figure 2:**
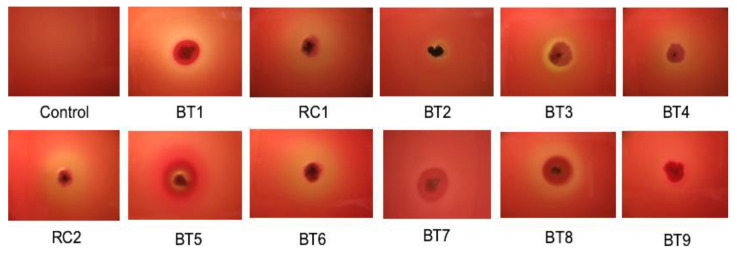
Actinobacterial isolates exhibiting varying degrees of haemolysis on 5% (*v*/*v*) blood agar plates after 7 days of incubation at 30 °C.

The majority of the actinobacterial isolates were able to produce lipase on agar media and exhibited biosurfactant activity. In addition, the strains produced haemolysins, exhibiting varying degrees of haemolysis when grown on horse blood agar (Figure 2).

### 3.4. Determination of Lipase Activity of Actinobacterial Isolates

Extensive preliminary testing of the strains for lipase activity using 4-NP as a substrate showed that: (i) the lipase activity was highest when strains were grown in broth containing tributyrin and olive oil, when compared with broth containing only tributyrin or olive oil; (ii) activity was greater at 37 **°**C than at 30 **°**C or 20 **°**C, except for strains RC2 and BT7 that did not grow at 37 **°**C; and (iii) activity reached a maximum between 5 and 10 days after incubation.

**Figure 3:**
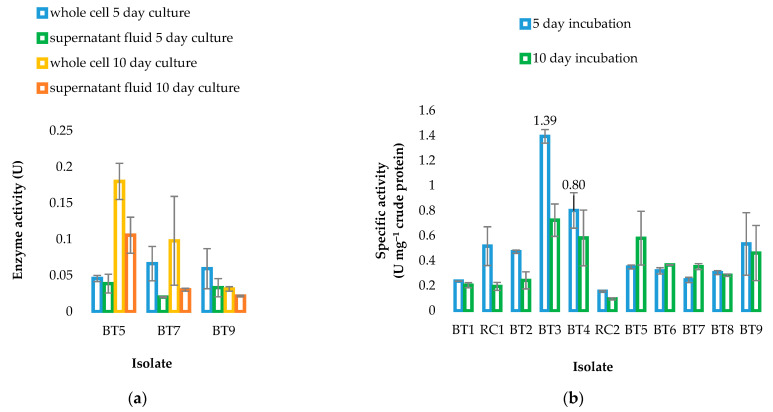
Differences in lipase activities in assays from whole cells and supernatant fluid (**a**), and specific lipase activities for all isolates (**b**) cultured in broth containing tributyrin and olive oil. Error bars represent standard deviation from the mean (*n* = 3).

Furthermore, significant differences were found in lipase activities between assays performed from whole cells and supernatant fluids (2-tailed *t*-test, two samples of equal variance: *p* < 0.05) in three isolates: BT5 (putative *Streptomyces setonii*, 10-day cultures only), BT7, and BT9 (putative *Gordonia jinhuaensis* (5 and 10 day cultures only, respectively)). Enzyme activity was notably higher in the assays from the whole cells (Figure 3a), strongly suggesting that these strains expressed intracellular or membrane lipase/s as well as extracellular lipase/s. Although extracellular lipases are well described in *Streptomyces* species, the specific presence of a whole cell lipase has only been reported in *Streptomyces clavuligerus* [39]. Only one report (the original strain type description) exists in the literature for *G. jinhuaensis*, in which the organism screened positive for lipase of unknown aetiology [40].

Following basic activity screening, the specific enzyme activities were determined using the optimized medium, as well as the optimized temperature for each isolate (30 **°**C or 37 **°**C). Two isolates (*Streptomyces* sp. BT3; *Streptomyces* sp. BT4) exhibited the highest activities (Figure 3b). The specific lipase activities of 0.80 and 1.39 U mg^−1^ crude protein for BT4 and BT3, respectively, are promising.

**Figure 4:**
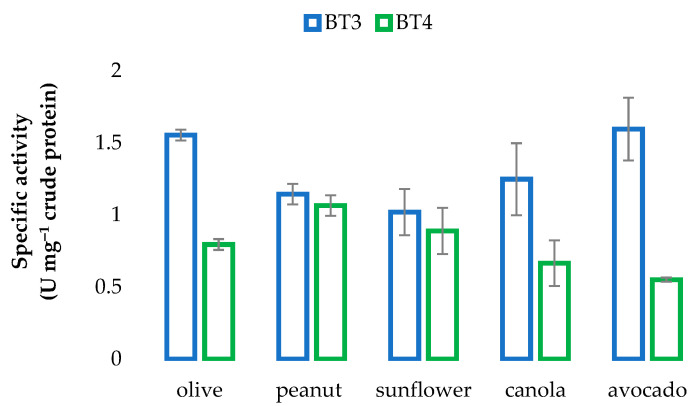
Comparision of enzyme actitivies of *Streptomyces* sp. BT3 and *Streptomyces* sp. BT4 determined after growth on a variety of oil-containing media. Error bars represent standard deviation from the mean (*n* = 3).

The lipase activities of BT3 and BT4 varied considerably after growth in media containing different oils (Figure 4). Notably: (i) from highest to lowest, the order of activity for BT3 and BT4 was avocado > olive > canola > peanut > sunflower, and peanut > sunflower > olive > canola > avocado, respectively; (ii) of all the oils tested, activities were highest for BT3 and lowest for BT4 when grown in media containing avocado oil; (ii) although the strains were isolated from an environment contaminated with canola oil, activity was not the highest for either strain when grown in media containing canola oil. These results reflected the different substrate preferences of the two strains.

The total fat content in the BT effluent (1.56%, *w*/*w*) was slightly higher than in the liquid culture media (0.71–0.84%, *w*/*w*). Of note was the fact that the type of fat (% saturated/unsaturated/poly-unsaturated) in the BT effluent was highly similar to that in the liquid culture medium containing canola oil.

When comparing the composition of canola oil with avocado oil (highest lipase activity for BT3) and peanut oil (highest lipase activity for BT4), it was noted that palmitic and palmitoleic acids were notably lower in canola than in avocado oil, while the concentrations of palmitic and linoleic acids were higher, and those of oleic and arachadic acids lower in peanut oil than in canola oil (Table 3). Previous studies have also found broad and varied fat/oil specificities for different *Streptomyces* species [41,42,43].

### 3.5. Genome Sequencing

Genome sequencing revealed that both strains BT3 and BT4 are members of the *Streptomyces albidoflavus* group. Members of this group have been isolated from diverse environments, including marine sponges, soil, deep-sea water, and leaf-cutter ants [44,45], with this study being the first to report the isolation of a *S. albidoflavus* from edible oil wastewater. Strain BT3 (6,847,979 bp) and BT4 (6,995,699 bp) both have a G + C% of 72.7% and are both closely related to *S. albidoflavus* J1074 (origin unknown) and *S. albidoflavus* S4 (symbiont of a leaf-cutter ant) [44]. An evaluation of their genome sequences revealed the presence of diverse lipases and esterases (see Supplementary Materials), which can mainly be classified as alpha/beta hydrolase, dienelactone hydrolase, serine hydrolase, lipase, esterase, and beta-lactamase. The majority of these enzymes contain a signal peptide that allows for secretion of the enzymes, with only the predicted dienelactone hydrolases and esterases containing a twin-arginine translocation signal peptide for the secretion of the protein (Supplementary Table S1).

AntiSMASH results showed the presence of diverse biosynthetic gene clusters (Supplementary Table S2). Notably, both strains have a high number of non-ribosomal peptide synthetase (NRPS) and polyketide synthase biosynthetic gene clusters. It is believed that these clusters are involved in the production of biosurfactants [46]. Predicted clusters for *Streptomyces* sp. BT3 show low levels of similarity to biosynthetic gene clusters involved in the production of known biosurfactants: glycinocin A (9%), candicidin (19%), pimaricin (23%), simocyclinone (5%), enduracidin (4%), and daptomycin (10%). Contrastingly, *Streptomyces* sp. BT4 only harbors a single biosynthetic gene cluster with similarity to the biosynthetic gene cluster involved in the production of friulimicin (9% similarity). The low sequence similarity may be due to an incomplete genome sequence, or it may indicate the potential of the strains to produce novel biosurfactants. Notably, most of these biosurfactants are cyclic lipopeptides or macrocyclic glycosides, two very important classes known for their potent antimicrobial activities [47].

Esterases and lipases are often produced in conjunction with lignocellulose-degrading enzymes. The dbCAN2 server makes use of different algorithms to predict the presence of carbohydrate degrading enzymes (CAzymes) and auxiliary enzymes involved in lignocellulose degradation [31]. Predicted CAzymes present in the genomes of strains BT3 and BT4 is summarized in Supplementary Table S3. It is not surprising that the two strains have similar CAzymes, with a few exceptions.

### 3.6. Microbiota Analysis for Selected Wastewater Streams

**Figure 5:**
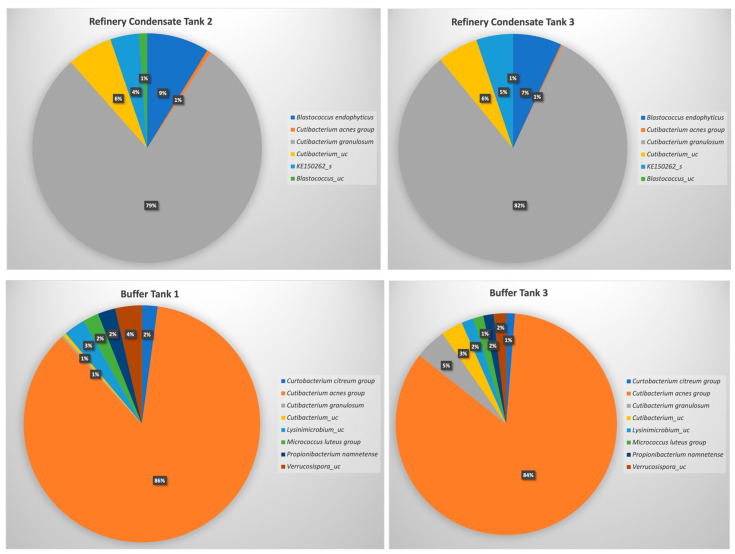
Comparative metagenomics of the actinobacterial populations of the refinery condensate tank (RT1 and RT2) and buffer tank (BT1 and BT2) grab samples. Only those with a relative abundance of >1% were included in this figure.

Microbial community analyses showed that all four grab samples were dominated by *Cutibacterium acnes* (previously classified as *Propionibacterium acnes*), representing >75% of the total community in all four samples (Figure 5). Despite this, the communities of the wastewater streams were vastly different and included diverse actinobacterial families (Figure 5). A comparison of the bacterial populations of the buffer tank (BT) and refinery condensate tank (RT) showed that the *C. acnes* group dominated both environments (Figure 5), with BT containing a greater diversity than RT. The greater degree of actinobacterial diversity was also clearly reflected in the α-diversity analyses (Table 4), with the buffer tank samples (BT1, BT2) exhibiting much higher values for the Shannon, Jackknife, and Chao1 indices, and lower values for the Simpson index. The lower number of valid reads, reads identified to species level, and number of OTUs can be ascribed to i) the physical conditions within the different tanks, or ii) the fact that the metagenomic DNA extraction was more efficient and less biased for the BT samples than for the RT samples (RT1, RT2). The latter explanation is more feasible, especially considering the higher CFUs obtained for this sample during the microbial isolation experiments (Figure 1). Non-specific amplification with the actinobacteria-specific primer set also resulted in the detection of Firmicutes and Proteobacteria, possibly also resulting in an underestimation of the actinobacterial diversity.

## 4. Discussion

Many types of industrial wastes contain oils or fats with varied chemical compositions of both polyunsaturated and saturated fatty acids. The composition ultimately determines whether microorganisms are able to utilize the particular fats/oils as substrates to grow and survive in these extreme environments [2]. Various actinobacterial strains were isolated from these nutrient-poor environments. It is possible that bacterial N-fixation may have compensated to some extent for the N deficiency in the wastewater, allowing bacterial proliferation in the effluent streams, as previously described [48]. The ability of actinobacterial strains to produce lipases and biosurfactants has previously been reported in literature and is, therefore, not surprising. Lipases and/or biosurfactants produced by *Streptomyces* species have been most widely published [4,41,42,47,49,50], with scattered reports for members of the genus *Arthrobacter* [51,52], *Gordonia* [47], and *Micromonospora* [47,53].

The studies reported here focused on a proof-of-concept idea that bacterial strains isolated from an oil-rich environment would have the ability to produce enzymes exhibiting lipase activities and have the ability to produce biosurfactants. Such future work would include increasing the activities by culture optimization, enzyme purification, and possibly gene expression in a heterologous host. Previous such studies where lipase activities were determined using 4-NP as a substrate have achieved high activities: under optimal growth conditions, [43] managed to achieve activities of 12.32 U mg^−1^ with *Streptomyces* sp. OC119.7 using filtered culture supernatant, which increased to 68.07 U mg^−1^ when purified using gel filtration; [41] achieved activities of 12.8 and 659.2 U mg^−1^ when using 15X concentrated culture filtrate and highly purified enzyme, respectively, with *Streptomyces rimosus*; [42] cloned and expressed the lipase gene SC111.14c from *Streptomyces coelicolor* A3(2) in the heterologous host, *Streptomyces lividans*; and increased the enzyme activity from 11 U mg^−1^ in the crude extract to 75.8 U mg^−1^ in a highly purified and concentrated form. Furthermore, the induction of biosurfactant production due to the presence of oils has been shown to positively impact on the production of lipases [54,55]. It is speculated that an increase in biosurfactants could result in an increase in cell membrane permeability and bioavailability of lipase substrates and subsequent secretion of lipases.

The two isolates that exhibited the highest lipase activity, BT3 and BT4, were identified to be closely related to *S. albidoflavus*. Analyses of the RAST-annotated genomes provided some insights into the types of lipases and esterases these strains can produce. Future studies would be focused on the cloning of the genes containing signal sequences that allow for extracellular activities. In conjunction, as indicated, the optimization of the growth conditions would not only provide insights into lipase/esterase production, but also for biosurfactant production, isolation, and characterization. In addition, the presence of various types of glycosyl transferases, glycoside hydrolases, carbohydrate esterases, and auxiliary proteins identified from CAzyme analyses, supports the potential of the strains to also degrade lignocellulosic material, highlighting the biotechnological potential of these *Streptomyces* strains.

To gain insight into the microbial community structure, including the relative abundance of the actinobacterial isolates in the samples, metagenomic DNA was extracted from the RC and BT samples and used in microbiota analyses. The genera that were isolated did not directly reflect the total actinobacterial population detected when using microbiota 16S rRNA gene sequence analyses, which may be due to bias from culture-based experiments and the ability/inability to identify diverse actinobacterial isolates on agar media. Surprisingly, all the samples analyzed were dominated by *C. acnes*. *Cutibacterium acnes* is the most common coloniser of human skin [56]. The ability to produce a range of lipolytic enzymes provides this lipophilic bacterium with a competitive advantage, allowing it to thrive in human sebaceous cysts [56,57]. Although, to our knowledge, the presence of this organism has not previously been reported in other ‘oily’ environments, it is likely that the metabolic capabilities of *C. acnes* would have allowed it to proliferate in the EO effluent.

A recent study by [58] showed that, in a clinical study, high numbers of *C. acnes* were detected in a range of samples analysed when using next generation sequencing. They concluded that the samples could have been contaminated at the source of collection (patients) or that the contamination stemmed from other sources, such as the environment or from laboratory reagents. Bearing in mind that the source of the organism could be the environment, a study by [59] reported that an inter-kingdom transfer may have occurred from humans to plants. This became evident when a unique phylotype of *C. acnes* was detected in the domesticated grapevine, *Vitis vinifera* L., showing that this endophyte most probably transferred to grapevines during the Neolithic period when grapevines were domesticated [59]. By studying the sequences of the *C. acnes* detected in this study, it was found that it was more closely related to *C. acnes* subsp. *defendens*, a subspecies of *C. acnes* also detected in the woody parts of grapevines [60]. Unlike *C. acnes* subsp. *acnes*, *C. acnes* subsp. *defendens* strains are not typically associated with clinical acne vulgaris but, as an opportunist, may cause skin infection [61,62]. Interestingly, other *Cutibacterium* species also detected in grapevines, *Cutibacterium namnetense* and *Cutibacterium granulosum*, were also detected in this study (Figure 5).

## 5. Conclusions

The main aim of this study was to determine whether wastewater generated in the canola oil industry could potentially serve as a source of lipolytic actinobacteria, especially since similar studies mostly focused on olive mill wastewater [63,64,65,66]. An analysis of the physicochemical properties of the wastewater showed that suitable macronutrients that would support the growth of microorganisms are found in wastewater generated in the canola oil industry. This was further supported by the high bacterial counts observed when an isolation approach was taken. Actinobacterial strains selected from isolation plates were screened for their ability to produce lipolytic enzymes and biosurfactants. Here, we demonstrated that various actinobacterial strains exhibited the ability to not only produce lipolytic enzymes, but also biosurfactants and/or haemolysins. It can therefore be concluded that novel actinobacteria with the ability to produce lipolytic enzymes and biosurfactants may be discovered in niche environments, such as canola oil industry wastewater. Notably, when cultivated in liquid media, different types of oils had varied effects on the lipase-producing ability of *Streptomyces* sp. strains BT3 and BT4, with increased activity being observed for strain BT3 in the presence of avocado and olive oil. It is possible that the presence of palmitoleic acid in these oils acted as an inducer for lipase production in this strain. To the best of our knowledge, the use of avocado oil as an inducer for the production of lipases from *Streptomyces* species have not been reported in the literature.

Microbiota analysis reflected the dominance of *C. acnes* subsp. *defendens* in the wastewater samples, with sequences related to those detected in plant-associated microbiomes, suggesting that this organism was either anthropogenically introduced or may be more ubiquitous in the environment than previously thought (for example, this organism may be found in association with the source plant material, *Brassica napus* subsp. *napus* used in the production of canola oil). The high CFU count from the RT samples indicates that microbiota analysis did not fully access the diversity associated with this sample, which can be expected since isolation approaches tend to introduce a bias for the isolation of specific microorganisms. Future studies will therefore focus on further optimization of the unique metagenomic DNA isolation protocol used in this study for the isolation of total DNA from wastewater contaminated with fats, oils, and grease, and the use of various isolation approaches. Strains BT3 and BT4 will be further analysed for their ability to produce lignocellulose-degrading enzymes and the application of these enzymes in synergy studies with the lipases produced by these strains.

## Figures and Tables

**Table 1:** Concentrations and ratios of major nutrients in wastewater samples.

	HE	AW	RC	BT	ST
COD screen (mg L^−1^)	45	ND *	8400	33,200	381,000
COD final (mg L^−1^)	ND	ND	<10	86,700	19,200
Total N final (mg L^−1^)	ND	ND	BDL	4	3
Total P final (mg L^−1^)	ND	ND	BDL	1290	1101
COD:N (ratio)	ND	ND	ND	21,625:1	6400:1
COD:P (ratio)	ND	ND	ND	67:1	17:1
FOG screen (mg L^−1^)	ND	110	1505	41,738	264,150
Total fat (%wt.wt)	ND	ND	0.21	1.56	0.23

HE = condensate from hexane extraction; WB = wash-bay; AW = acid wastewater; RC = refinery condensate; BT = buffer tank; ST = separator tank; COD = chemical oxygen demand; FOG = fats, oils and grease; ND = not determined; BDL = below detection limit of analytical method; * chemical interference with analytical method.

**Table 2:** Results of screening of actinobacterial isolates for lipase and biosurfactant activities.

Strain	Nearest Phylogenetic Neighbour (% Sequence Similarity)	TDA	EYA	TBA	OOR	DC	OS (Zone)	E24
BT1	*Arthrobacter ruber* MDB1-42 (99.12%)	+	+	+	−	+ CMO *	+ ** (0.5 cm)	−
RC1	*Streptomyces werraensis* NBRC 13404 (99.50%)	+	+	+	−	+ CMO	−	68%
BT2	*Micromonospora fluminis* A38 (99.27%)	+	+	+	−	+ M	−	−
BT3	*Streptomyces albidoflavus* DSM 40455 (99.77%)	+	+	+	+	+ CM *	−	−
BT4	*Streptomyces albidoflavus* DSM 40455 (99.64%)	+	+	+	+	+ CM *	−	−
RC2	*Streptomyces pratensis* ch24 (99.49%)	+	+	+	−	+ CMO *	−	62% **
BT5	*Streptomyces setonii* NRRL ISP-5322 (99.78%)	+	+	+	+	+ M	−	88%
BT6	*Streptomyces colonosanans* MUSC 93J (98.00%)	−	+	+	−	+ M	−	60% **
BT7	*Gordonia jinhuaensis* ZYR 51 (99.49%)	+	+	+	+	+ CMO *	+ ** (1 cm)	60% **
BT8	*Streptomyces griseoincarnatus* LMG 19316 (98.28%)	+	−	+	−	+ CM *	−	−
BT9	*Gordonia jinhauensis* ZYR 51 (98.99%)	+	−	+	+	+ CMO *	+ ** (4 cm)	60% **

TDA = tween degradation agar; EYA = egg yolk agar; TBA = tributyrin agar; OOR = olive oil agar with Rhodamine; BT = buffer tank; RC = refinery condensate; DC = drop collapse; OS = oil spreading; E24 = emulsification index; M = mineral oil; C = canola oil; O = olive oil; * drop collapse by strains cultivated in YEME supplemented with 0.1% (*v*/*v*) canola oil; ** strains cultivated in YEME supplemented with 0.1% (*v*/*v*) canola oil—other positive strains were cultivated in YEME.

**Table 3:** Fats and fatty acids profiles in wastewater and edible oil samples.

Type of Fat (% of Total Fat in Sample)										Total Fat (%, *w*/*w* of Sample)		
	Saturated			Unsaturated			Poly-Unsaturated					
Avocado	20.8			68.6			10.6			83.98 (0.84) *		
Olive	17.6			78.1			4.3			81.37 (0.81) *		
Peanut	20.8			42.7			36.5			71.44 (0.71) *		
Sunflower	13.3			33.1			53.7			73.35 (0.73) *		
Canola	20.7			62.1			17.2			78.44 (0.78) *		
RC	28.6			47.6			23.8			0.21		
BT	23.7			57.1			19.2			1.56		
ST	30.4			47.8			21.7			0.23		
**Fatty acid type (%, *w*/*w* of sample)**												
	**Palmitic**		**Stearic**		**Arachidic**		**Palmitoleic**		**Oleic**		**Linoleic**	
Avocado	15.96		0.80		0.73		4.45		53.12		8.92	
Olive	10.90		3.45		0		0.76		62.75		3.51	
Peanut	11.33		3.56		0		0		30.49		26.06	
Sunflower	6.07		3.67		0		0		24.25		39.36	
Canola	4.81		2.20		9.26		0		48.69		13.48	

* Total fat in culture medium; RC = refinery condensate; BT = buffer tank; ST = separator tank; results are averages of duplicate determinations.

**Table 4:** Comparision of the microbial population data for the buffer tank (BT1 and BT2) and refinery condensate tank (RT1 and RT2) grab samples.

	BT1	BT2	RT1	RT2
**Parameter**				
Total valid reads	57,271	25,339	1711	1035
Number of reads identified to species level	30,640	17,544	1484	898
Number of species found (97% cut-off)	130	97	21	20
Average read lengths (bp)	237.6	236.8	228.6	229.8
**α-diversity**				
Shannon	2.246	2.049	1.322	1.226
Jackknife	410	258	35.0	32.0
Chao1	373.8	236.3	32.0	29
Simpson	0.247	0.342	0.507	0.547
Number of OTUs found in sample	363	226	29	26

## Data Availability

GenBank assembly accession numbers have been assigned to both genomes reported in this study: BT3—GCA_016734865.1 (BioSample SAMN14167118) and BT4—GCA_016734825.1 (BioSample SAMN14167119). Accession numbers have also been assigned to all 16S rRNA gene sequences reported in this study and are available from GenBank (accession numbers: MZ889014-MZ889024). Raw sequence data generated from the amplicon-based metagenomics study are available from NCBI under the accession number PRJNA757224.

## Supplementary Materials

The following are available online at https://www.mdpi.com/article/10.3390/microorganisms9091987/s1. Table S1: Lipases and esterases detected within the annotated genomes of BT3 and BT4. Export signals were detected through SignalP-5.0 analysis. Table S2: Biosynthetic gene clusters detected within the genome sequences of *Streptomyces* sp. BT3 and *Streptomyces* sp. BT4. Table S3: CAzymes predicted to be present in the genomes of *Streptomyces* sp. BT3 and *Streptomyces* sp. BT4 (predicted by dbCAN2). Only CAzymes predicted by all three algorithms are listed.
