# Supplementary material for: Wastewater from the Edible Oil Industry as a Potential Source of Lipase- and Surfactant-Producing Actinobacteria
Source: Microorganisms. 2021 Sep 18;9(9):1987. doi: 10.3390/microorganisms9091987 (PMC8465459; doi:10.3390/microorganisms9091987)
Supplement: Supplementary file 1 [file microorganisms-09-01987-s001.zip › microorganisms-1381239-supplementary.pdf]

Wastewater from the edible oil industry as a potential source of lipase- and surfactant-producing actinobacteria

Pamela Welz [1], Gustav Swanepoel [1], Shandré Weels[1] and Marilize Le Roes-Hill [1,*]

[1] Applied Microbial and Health Biotechnology Institute, Cape Peninsula University of Technology, PO Box 1906, Bellville, 7535, South Africa; WelzP@cput.ac.za; swangustav@gmail.com; shandrelavern@gmail.com; LeroesM@cput.ac.za
* Correspondence: Leroesm@cput.ac.za; Tel.: +27 21 953 8499

Supplementary information

Lipase and esterase sequences annotated for strains BT3 and BT4

>BT3_putative_lipase_esterase
MGSSRASRPGRGRAGASRTLRGPCTAEQGPAGPGVAPGPGPSWAGPGPGQGPTGPQYGRAEAAS
RAEEDDGGRSAERGGARGGGGELPVRAPGGGAGHHGGVRPGPGPGGGLLRPARSGRGTGHAAG
RGGARRGVAGRARPAAHLAVRGVPGGARVRGGERGVPAGQRRAGRRALADTFDDIAAAFDALP
ELAAEAVPEADPGRTVLTGHSAGGHLALWAAARHVLPAGSPWRLPEAPAVRGVVALAPIADFAT
SAELNVCGGAFAQLLGEEYEERLAHADPTALLPTGMATTLVQGTVDTEVPPAVAEAYADAATAA
GETPGFTLVDGVGHFPLIDPASAACALVAEEIAQLAW

>BT3_putative_esterase
MSTMWQGCLAHTDYFEMRSGGGHDYGIWVTTPPGYDAAVAQAPVVYVLDGNWTVGLTAPLIVT
QLDPMQSIRPYIQVSVGYAGKEAEHWERLRNRDLVPPGEPIAKEYVDAVEMSVETGTTTRDQADA
YLAELSNSRGDMFLDFLTDDLHPRIARDWGAAPNGHGLFGYSYGGLFSLYAWLTRSTFFESVGAGS
PGVVDTSSQVFARLQAMGDALPATRLHVTFNERELLGDIAVYQNLAKNAATFLHRLTARGGPVTS
ALLHETHVTGLQASFLSYLRTCRAQ

>BT3_lipase
MQSTPRTGTTTPPPTRRTRTRRLAGTAAAVAAVVSLTTLSTPGAHAADNPYERGPAPTRASIEAPRG
PYAVSQTSVSSLLVSGFGGGTIYYPTSTSDGTFGAVVVTPGFTATESSMAWLGPRLASQGFVVFTIDT
LTTLDQPDSRGRQMLAALDYLTERSSARTRIDGTRLGVIGHSMGGGGTLEAAKSRPSLKAAIPLTPW
NLDKTWPEVTTPTLVVGADGDTVAPVATHAKPFYSTLPSTTDRAYLELNNATHFAPNISNTTIAKY
SVSWLKRFIDDDTRYEQFLCPLPEPDRDIEEYRGTCPSVADGRPLARPKWRTARFRTGLRTRNLPGM
TGEESALPTVRLHGRAPQLSRIRAWWHQPPAHRSALLVAGEPGLGRTAVLRWAARALGADSTGH
LVAGPAPA

>BT3_esterase_lipase
MPAPCLVWFHGGGMVLGTPETDDARVSAYARDVGCVVVSAEYRPAPEHPHPVPVEDCYRALGW
TAGQAKALGVDPHRLAVGGISAGGGLAAATALLARDRGGPALAFQLLLCPMLDDRNTTPSSREFS
RAVAWPRADNLFGWSALLGPLAGTGRVPPHAAPARAADLSGLPAASVDVGELEVFRDECARYAL
RLAEAGVPAELRLSPGAFHGFDGILPQVTLSRRAAAEQVAALRRALGGC

>BT3_putative_esterase

MNDTAEATGHPAPDEQDPAPEPVAVHGTVAPGYEPVRDAFARNFAQLGDRGAAVTVHRHGSPV
VDLWAGTRDHDSTAPWERGTAQVVHSVTKGVSAAALLLLHQRGRLDLDAPVSTYWPEYAAEGK
ERTLVRHLLAHRAGVPVLDRPLTPDEAAHPGTGAAAVAAQAPVWEPGTEHGYHAQTYSWLTGE
LVRRVTGISLGRYVAEEIAGPLGLDLWIGLPPAQKDRVGRSGAVSAPERGGGLRLRPRRNVQDAYG
DPASLTRRAFDAVDPPADENSPAYHAAELPGANGIATAYALSRFYASLIGAVEQGPRLFAPATLAL
ARTVESEGPDRVLVVPTRFGPGFMLHGAASPLLAEGSFGHPGRGGSLGFADPESGVAFGYVTNGLQ
KTVTNDPRAQALVRAVRGVLQG

>BT3_putative_lipase
MASQLPPPPDPYLETAAKELAEATDPHPRIYEVPPAQGRDILAGLQSGEGVERPEVDEEWVTVDAG
QWGQVRTRIIRPKGATGPLPVVFYIHGAGWVFGDENTHDRLFRELTVGAGAAGVFPVYDRAPEAQ
YPTQVEQNYAVGQWVLQRGAEHGLDTSRIAVTGESVGGAMSAVFAIMNKERGGIDLKGQVLLYP
VANADFGTPSYLQFAEGYYLTRDGMKWFWDQYAPNPAHRTEVYASPLQATTEQLRGLPPALVVT
DEADVLRDEGEAYAARLREAGVDVTAVRVAGMVHDFLLLDSLRDTRAANVARHLAIDALTKAL
HD

>BT3_putative_lipase
MLYSPGLQVSRTLGTATAIELASRGYVVVAVDHTYEAPAVEFPGGRVEPQQPMSGTGDLKKMVET
RVGDTRFVLDQLATGRGLPERLGKALDLERVGMYGHSGGGATAAEAMRVDRRIDAGINMDGTLQ
YDDTDFLPVAREGLDRPFMLMGKPGQSHLVKASWRSFWDRSTGWKRDLSLTRGSHFSYTDAQSFV
PALDEHLDIPGPLREQYIGTVDPVRSTAAQRAYIAAFFDQHLRQRPQELLDGPSAAHPEVRFVD

>BT3_putative_esterase
MSTSRPTRRGVLKAAAGLTATTALGATSVLASASAAHAAGDGFGLRIVDRNESDARMWYYRFAT
DAIGWDPGVNVLLPDGYHAGGRRYPVLYLFHGGGTGQDFITFDRMGIRAWTAGKPLIVVMPDGG
PAGWYSNPVSSNVGPRNWETFHIAQLLPWIDANFRTYAEYDGRAVSGFSMGGFGALKYAAKYYG
HFASVSSHSGPASLRRDAGLVTHWANVSSAAVELGGGTVYGAPLWDEARVSADNPVQRVESYRN
KRVFLVAGTSPDPVNWFDTVNETQVLAGQREFKSVLGAAGIPHEGHEVPGGHFVRPDLFIRDLDGI
VARLRKA

>BT3_putative_esterase
MCTVEIGGWADEGFGGVADVFRENFDVFGELGTAVSVRAGGRPVVELWGGVADERSGRPWDQE
TVVPVFSCAKGLVSICAHLLAQQGRLDLDAPVAAYWPEFAAEGKERITTRMVLGHRAGVPVLDRT
VSFGEITEWTPVVRAIEEQRPLWEPGEAYEYHGHVFGFLVGEVIRRITGLTPGRFFREAVGGPLGLRT
WIGLPAAERGKLARLVEAEGRPAGDPQSLLMRIVTMNGALVFPGLEEPHGFNDPELLGTELPGAG
AVASASGLAALYGAAVTGLDGGPRLLSEETLTDAVREVSAGPTWQGYDLGQRWGSGFLLDSERPR
PMLGGRSFGNDGAGGQFAFGDDEFGVGFAYVANRMIGHGDDRANRLITALRGSLKA

>BT3_putative_esterase_lipase
MPVLPGAEPYRHDGGPTGVLLCHGFTGTPQSMRPWAEDLAARGHTVSLPLLPGHGTRWEDLQLT
GWQDWYAEVDRALGELLARCERVYVAGLSMGGALALRLAARHGDAVRGVVVVNPANKVHGLS
AYALPAARHLVRTTKGLVSDIALEGSEEVGYHRVPLHAAHSLRNFFRLVDAELPQVTQPLLLLRSPR
DHVVPPADSARVLGRVSSRDVTEILLQNSFHVATLDHDAPRVFEESAAFLARTGANGADSRPEGEA
VSG

>BT4_putative_esterase
MSTMWQGCLAHTDYFEMRSGGGHDYGIWVTTPPGHDAAVAQAPVVYVLDGNWTVGLTAPLIVT
QLDPMQSIRPYIQVSVGYAGKEAEHWERLRNRDLVPPGEPIAKEYVDAVEMSVETGTTTRDQADA
YLAELSNSRGDMFLDFLTDDLHPRIARDWGAAPNGHGLFGYSYGGLFSLYAWLTRSTFFESVGAGS

PGVVDTSSQVFARLQAMGDALPATRLHVTFNERELLGDIAVYQNLAKNAATFLHRLTARGGPVTS
ALLHETHVTGLQASFLSYLRTCRAQ

>BT4_putative_esterase
MCTVEIGGWADEGFGGVADVFRENFDVFGELGTAVSVRAGGRPVVELWGGVADERSGRPWDQE
TVVPVFSCAKGLVSICAHLLAQQGRLDLDAPVAAYWPEFAAEGKERITTRMVLGHRAGVPVLDRT
VSFGEITEWTPVVRAIEEQRPLWEPGEAYEYHGHVFGFLVGEVIRRITGLTPGRFFREAVGGPLGLRA
WIGLPAAERGKLARLVEAEGRPAGDPQSLLMRIVTMNGALVFPGLEEPHGFNDPELLGTELPGAG
AVASASGLAALYGAAVTGLDGGPRLLSEETLTDAVREVSAGPTWQGYDLGQRWGSGFLLDSGRPR
PMLGGRSFGNDGAGGQFAFGDDEFGVGFAYVANRMIGHGDDRANRLITALRGSLKA

>BT4_putative_lipase_esterase
MGAARSAAEREAAEESSLFGHPEAAPDTTVAYGPDPDQVVDFYAPRGPGAAPGTPLVVVVHGGA
WRAAHDRLHISPFAAFLAGRGFAVASVEYRRGNGGQVAGRWPDTFDDIAAAFDALPELAAEAVP
EADPGRTVLTGHSAGGHLALWAAARHVLPAGSPWRLPEAPAVRGVVALAPIADFATSAELNVCG
GAFAQLLGEEYEERLAHADPTALLPTGMATTLVQGTVDTEVPPAVAEAYADAATAAGETPGFTLV
DGVGHFPLIDPASAACALVAEEIAQLAW

>BT4_lipase
MQSTPRTGTTTPPPTRRTRTRRLAGTAAAVAAVVSLTTLSTPGAHAADNPYERGPAPTRASIEAPRG
PYAVSQTSVSSLLVSGFGGGTIYYPTSTSDGTFGAVVVTPGFTATESSMAWLGPRLASQGFVVFTIDT
LTTLDQPDSRGRQMLAALDYLTERSSARTRIDGTRLGVIGHSMGGGGTLEAAKSRPSLKAAIPLTPW
NLDKTWPEVTTPTLVVGADGDTVAPVATHAKPFYSTLPSTTDRAYLELNNATHFAPNISNTTIAKY
SVSWLKRFIDDDTRYEQFLCPLPEPDRDIEEYRGTCPSVADGRPLARPKWRTARFRTGLRTRNLPGM
TGEESALPTVRLHGRAPQLSRIRAWWHQPPAHRSALLVAGEPGLGRTAVLRWAARALGADSTGH
LVAGRRRPPHQDV

>BT4_esterase_lipase
MVSAEYRPAPEHPHPVPVEDCYRALGWTAGQAKALGVDPHRLAVGGISAGGGLAAATALLARD
RGGPALAFQLLLCPMLDDRNTTPSSREFSRAVAWPRADNLFGWSALLGPLAGAGRVPPHAAPAR
AADLSGLPAASVDVGELEVFRDECARYALRLAEAGVPAELRLSPGAFHGFDGALPQVTLSRRAAA
EQVAALRRALGGC

>BT4_putative_esterase
MSTSRPTRRGVLKAAAGLTATTALGATSVLASASAAHAAGDGFGLRIVDRNESDARMWYYRFAT
DAIGWDPGVNVLLPDGYHAGGRRYPVLYLFHGGGTGQDFITFDRMGIRAWTAGKPLIVVMPDGG
PAGWYSNPVSSNVGPRNWETFHIAQLLPWIDANFRTYAEYDGRAVSGFSMGGFGALKYAAKYYG
HFASVSSHSGPASLRRDAGLVTHWANVSSAAVELGGGTVYGAPLWDEARVSADNPVQRVESYRN
KRVFLVAGTSPDPVNWFDTVNETQVLAGQREFKSVLGAAGIPHEGHEVPGGHFVRPDLFIRDLDGI
VARLRKA

>BT4_putative_esterase_lipase
MPVLPGAEPYRHDGGPTGVLLCHGFTGTPQSMRPWAEDLAARGHTVSLPLLPGHGTRWEDLQLT
GWQDWYAEVDRALGELLARCERVYVAGLSMGGALALRLAARHGDAVRGVVVVNPANKVHGLS
AYALPAARHLVRTTKGLVSDIALEGSEEVGYHRVPLHAAHSLRNFFRLVDAELPQVTQPLLLLRSPR
DHVVPPADSARVLGRVSSRDVTEILLQNSFHVATLDHDAPRVFEESAAFLARTGANGADSRPEGEA
VSG

>BT4_putative_esterase
MNDTAEATGHPAPDEQDPAPEPVAVHGTVAPGYEPVRDAFARNFAQLGDRGAAVTVHRHGSPV
VDLWAGTRDHDSTAPWERGTAQVVHSVTKGVSAAALLLLHQRGRLDLDAPVSTYWPEYAAEGK
ERTLVRHLLAHRAGVPVLDRPLTPDEAAHPGTGAAAVAAQAPVWEPGTEHGYHAQTYSWLTGE
LVRRVTGISLGRYVAEEIAGPLGLDLWIGLPPAQKDRVGRSGAVSAPERGGGLRLRPRRNVQDAYG
DPASLTRRAFDAVDPPADENSPAYHAAELPGANGIATAYALSRFYASLIGAVEQGPRLFAPATLAL
ARTVESEGPDRVLVVPTRFGPGFMLHGAASPLLAEGSFGHPGRGGSLGFADPESGVAFGYVTNGLQ
KTVTNDPRAQALVRAVRGVLQG

>BT4_putative_lipase
MLYSPGLQVSRTLGTATAIELASRGYVVVAVDHTYEAPAVEFPGGRVEPQQPMSGTGDLKKMVET
RVGDTRFVLDQLATGRGLPERLGKALDLERVGMYGHSGGGATAAEAMRVDRRIDAGINMDGTLQ
YDDTDFLPVAREGLDRPFMLMGKPGQSHLVKASWRSFWDRSTGWKRDLSLTRGSHFSYTDAQSFV
PALDEHLDIPGPLREQYIGTVDPVRSTAAQRAYIAAFFDQHLRQRPQELLDGPSAAHPEVRFVD

>BT4_putative_lipase
MASQLPPPPDPYLETAAKELAEATDPHPRIYEVPPAQGRDILAGLQSGEGVERPEVDEEWVTVDAG
QWGQVRTRIIRPKGATGPLPVVFYIHGAGWVFGDENTHDRLFRELTVGAGAAGVFPVYDRAPEAQ
YPTQVEQNYAVGQWVLQHGAEHGLDTSRIAVTGESVGGAMSAVFAIMNKERGGIDLKGQVLLYP
VANADFGTPSYLQFAEGYYLTRDGMKWFWDQYAPNPAHRTEVYASPLQATTEQLRGLPPALVVT
DEADVLRDEGEAYAARLREAGVDVTAVRVAGMVHDFLLLDSLRDTRAANVARHLAIDALTKAL
HD

Supplementary Table S1: Lipases and esterases detected within the annotated genomes of BT3 and BT4. Export signals were detected through SignalP-5.0 analysis.

Strain	Lipase/Esterase	BLASTp top hit	SignalP likelihood
BT3	Putative lipase/esterase	*Streptomyces* alpha/beta hydrolase	0.67 (other)
	Putative esterase/lipase	*Streptomyces* alpha/beta hydrolase	0.97 (other)
	Lipase	*Streptomyces* dienelactone hydrolase	0.60 (TAT)
	Esterase/lipase	*Streptomyces* alpha/beta hydrolase	0.70 (other)
	Putative esterase	*Streptomyces* serine hydrolase	0.96 (other)
	Putative lipase	*Streptomyces* alpha/beta hydrolase	0.98 (other)
	Putative lipase	*Streptomyces* lipase	0.88 (other)
	Putative esterase	*Streptomyces* esterase	0.93 (TAT)
	Putative esterase	*Streptomyces* beta-lactamase	0.98 (other)
	Putative esterase	*Streptomyces* alpha/beta hydrolase	0.96 (other)
BT4	Putative esterase	*Streptomyces* alpha/beta hydrolase	0.97 (other)
	Putative esterase	*Streptomyces* beta-lactamase	0.98 (other)
	Putative lipase/esterase	*Streptomyces* alpha/beta hydrolase	0.96 (other)
	Lipase	*Streptomyces* dienelactone hydrolase	0.60 (TAT)
	Esterase/lipase	*Streptomyces* alpha/beta hydrolase	0.82 (other)
	Putative esterase	*Streptomyces* esterase	0.93 (TAT)
	Putative esterase/lipase	*Streptomyces* alpha/beta hydrolase	0.97 (other)
	Putative esterase	*Streptomyces* serine hydrolase	0.96 (other)
	Putative lipase	*Streptomyces* lipase	0.88 (other)
	Putative lipase	*Streptomyces* alpha/beta hydrolase	0.98 (other)

TAT = Twin arginine translocation signal peptide

Supplementary Table S2: Biosynthetic gene clusters detected within the genome sequences of *Streptomyces* sp. BT3 and *Streptomyces* sp. BT4.

**Biosynthetic gene cluster**	**BT3 (number of clusters present)**	**BT4 (number of clusters present)**
Butyrolactone	1	1
Ectoine	1<br><br>1x 100% match: ectoine	1<br><br>1x 100% match: ectoine
Lanthipeptide class III	2	2
Lassopeptide	1	1
Linear azol(in)e-containing peptides (LAP)	1	1
Non-ribosomal peptide synthetase (NRPS)	20<br><br>1x 100% match: rhizomide A/B/C	16
NRPS-like	4	3
NRPS/RRE-containing	-	1
NRPS/T1PKS	3	2
RRE-element containing cluster (RRE-containing)	2	-
Other unspecified ribosomally synthesised and post-translationally modified peptide product (RiPP) cluster (RiPP-like)	2	2
Siderophore	3<br><br>1x 100% match: desferrioxamin	3<br><br>1x 100% match: desferrioxamin
Terpene	8<br><br>1x 100% match: geosmin	6<br><br>1x 100% match: geosmin
Trans-AT-PKS	-	1
Type 1 Polyketide synthase (T1PKS)	23<br><br>1x 100% match: 1-heptadecene	24<br><br>1x 100% match: 1-heptadecene
T1PKS/terpene	-	1
Type 3 Polyketide synthase (T3PKS)	1	1

Supplementary Table S3: CAzymes predicted to be present in the genomes of *Streptomyces* sp. BT3 and *Streptomyces* sp. BT4 (predicted by dbCAN2). Only CAzymes predicted by all three algorithms are listed.

**CAzymes predicted**	***Streptomyces* sp. BT3**	***Streptomyces* sp. BT4**
Auxialiary activity family 3<br>cellobiose dehydrogenase; glucose 1-oxidase; aryl alcohol oxidase; alcohol oxidase; pyranose oxidase	AA3 (1)	AA3 (1)
Auxiliary activity family 10<br>Copper-dependent lytic polysaccharide monooxygenases (LPMOs); some proteins have been shown to act on chitin, others on cellulose; lytic cellulose monooxygenase (C1-hydroxylating); lytic cellulose monooxygenase (C4-dehydrogenating); lytic chitin monooxygenase; lytic xylan monooxygenase / xylan oxidase (glycosidic bond-cleaving)	AA10 (3)	AA10 (3)
Carbohydrate-binding module family 2<br>This module has been found to bind cellulose, chitin or xylan	CBM2 (2)	CBM2 (2)
Carbohydrate-binding module family 3<br>Binds to cellulase; one report of binding to chitin	CBM3 (1)	CBM3 (1)
Carbohydrate-binding module 6<br>The cellulose-binding function has been demonstrated in one case on amorphous cellulose and β-1,4-xylan. Some of these modules also bind β-1,3-glucan, β-1,3-1,4-glucan, and β-1,4-glucan.	CBM6 (2)	CBM6 (2)
Carbohydrate-binding module 13<br>Often found in association with xylanase	CBM13 (4)	CBM13 (4)
Carbohydrate-binding module family 20<br>Starch-binding CBM, strongly interacts with cyclodextrins	CBM20 (1)	CBM20 (1)
Carbohydrate-binding module family 32<br>Binds galactose and lactose, polygalacturonic acid, and LacNAc (β-D-galactosyl-1,4-β-D-N-acetylglucosamine)	CBM32 (3)	CBM32 (2)

**CAzymes predicted**	***Streptomyces* sp. BT3**	***Streptomyces* sp. BT4**
Carbohydrate-binding module family 35<br>Binds to xylan, mannooligosacchardies, β-galactan	CBM35 (1)	CBM35 (1)
Carbohydrate-binding module family 42<br>Found mostly at the C-terminus of GH54 catalytic domains; binds to arabinofuranose	CBM42 (1)	CBM42 (1)
Carbohydrate-binding module family 48<br>Glycogen-binding module usually in association with GH13 modules	CBM48 (1)	CBM48 (2)
Carbohydrate esterase family 1<br>acetyl xylan esterase; cinnamoyl esterase; feruloyl esterase; carboxylesterase; S-formylglutathione hydrolase; diacylglycerol O-acyltransferase; trehalose 6-O-mycolyltransferase	CE1 (1)	CE1 (1)
Carbohydrate esterase family 4<br>acetyl xylan esterase; chitin deacetylase; chitooligosaccharide deacetylase; peptidoglycan GlcNAc deacetylase; peptidoglycan N-acetylmuramic acid deacetylase	CE4 (3)	CE4 (3)
Carbohydrate esterase family 7<br>acetyl xylan esterase; cephalosporin-C deacetylase	CE7 (1)	CE7 (1)
Carbohydrate esterase family 9<br>N-acetylglucosamine 6-phosphate deacetylase; N-acetylglucosamine 6-phosphate deacetylase	CE9 (1)	CE9 (1)
Carbohydrate esterase family 14<br>N-acetyl-1-D-myo-inosityl-2-amino-2-deoxy-α-D-glucopyranoside deacetylase; diacetylchitobiose deacetylase; mycothiol S-conjugate amidase	CE14 (2)	CE14 (2)
Glycoside hydrolase family 1	GH1 (4)	GH1 (4)

**CAzymes predicted**	***Streptomyces* sp. BT3**	***Streptomyces* sp. BT4**
β-glucosidase; β-galactosidase; β-mannosidase; β-glucuronidase; β-xylosidase; β-D-fucosidase; phlorizin hydrolase; exo-β-1,4-glucanase; 6-phospho-β-galactosidase; 6-phospho-β-glucosidase; strictosidine β-glucosidase; lactase; amygdalin β-glucosidase; prunasin β-glucosidase; vicianin hydrolase; raucaffricine β-glucosidase; thioglucosidase; β-primeverosidase; isoflavonoid 7-O-β-apiosyl-β-glucosidase; ABA-specific β-glucosidase; DIMBOA β-glucosidase; β-glycosidase; hydroxyisourate hydrolase; β-rutinosidase /α-L-rhamnose-(1,6)-β-D-glucosidase		
Glycoside hydrolase family 2<br>β-galactosidase; β-mannosidase; β-glucuronidase; α-L-arabinofuranosidase; mannosylglycoprotein endo-β-mannosidase; exo-β-glucosaminidase; α-L-arabinopyranosidase; β-galacturonidase; β-xylosidase; β-D-galactofuranosidase; β-glucosidase	GH2 (2)	GH2 (2)
Glycoside hydrolase family 3<br>β-glucosidase; xylan 1,4-β-xylosidase; β-glucosylceramidase; β-N-acetylhexosaminidase; α-L-arabinofuranosidase; glucan 1,4-β-glucosidase; isoprimeverose-producing oligoxyloglucan hydrolase; coniferin β-glucosidase; exo-1,3-1,4-glucanase; β-N-acetylglucosaminide phosphorylases; β-1,2-glucosidase; β-1,3-glucosidase; xyloglucan-specific exo-β-1,4-glucanase / exo-xyloglucanase	GH3 (5)	GH3 (6)
Glycoside hydrolase family 4<br>maltose-6-phosphate glucosidase; α-glucosidase; α-galactosidase; 6-phospho-β-glucosidase; α-glucuronidase; α-galacturonase; palatinase	GH4 (2)	GH4 (2)
Glycoside hydrolase family 5	GH5 (0)	GH5 (1)

**CAzymes predicted**	***Streptomyces* sp. BT3**	***Streptomyces* sp. BT4**
endo-β-1,4-glucanase / cellulase; endo-β-1,4-xylanase; β-glucosidase; β-mannosidase; β-glucosylceramidase; glucan β-1,3-glucosidase; exo-β-1,4-glucanase / cellodextrinase; glucan endo-1,6-β-glucosidase; mannan endo-β-1,4-mannosidase; cellulose β-1,4-cellobiosidase; steryl β-glucosidase; endoglycoceramidase; chitosanase; β-primeverosidase; xyloglucan-specific endo-β-1,4-glucanase; endo-β-1,6-galactanase; β-1,3-mannanase; arabinoxylan-specific endo-β-1,4-xylanase; mannan transglycosylase; lichenase / endo-β-1,3-1,4-glucanase; β-glycosidase; endo-β-1,3-glucanase / laminarinase; β-N-acetylhexosaminidase; chitosanase; β-D-galactofuranosidase; β-galactosylceramidase; β-rutinosidase /α-L-rhamnose-(1,6)-β-D-glucosidase; α-L-arabinofuranosidase; glucomannan-specific endo-Î²-1,4-glucanase		
Glycoside hydrolase family 6<br>endoglucanase; cellobiohydrolase; lichenase / endo-β-1,3-1,4-glucanase	GH6 (4)	GH6 (3)
Glycoside hydrolase family 10<br>endo-1,4-β-xylanase; endo-1,3-β-xylanase; tomatinase; xylan endotransglycosylase; endo-β-1,4-glucanase	GH10 (1)	GH10 (1)
Glycoside hydrolase family 13	GH13 (10)	GH13 (10)

**CAzymes predicted**	***Streptomyces* sp. BT3**	***Streptomyces* sp. BT4**
α-amylase; pullulanase; cyclomaltodextrin glucanotransferase; cyclomaltodextrinase; trehalose-6-phosphate hydrolase; oligo-α-glucosidase; maltogenic amylase; neopullulanase; α-glucosidase; maltotetraose-forming α-amylase; isoamylase; glucodextranase; maltohexaose-forming α-amylase; maltotriose-forming α-amylase; branching enzyme; trehalose synthase; 4-α-glucanotransferase; maltopentaose-forming α-amylase; amylosucrase; sucrose phosphorylase; malto-oligosyltrehalose trehalohydrolase; isomaltulose synthase; malto-oligosyltrehalose synthase; amylo-α-1,6-glucosidase; α-1,4-glucan: phosphate α-maltosyltransferase; amino acid transporter; [retaining] sucrose 6(F)-phosphate phosphorylase; [retaining] glucosylglycerol phosphorylase; Glucosylglycerate phosphorylase; [retaining] sucrose α-glucosidase; oligosaccharide α-4-glucosyltransferase; [retaining] α-amylase		
Glycoside hydrolase family 15<br>glucoamylase; glucodextranase; α,α-trehalase; dextran dextrinase	GH15 (2)	GH15 (1)
Glycoside hydrolase family 16<br>xyloglucan:xyloglucosyltransferase; keratan-sulfate endo-1,4-β-galactosidase; endo-1,3-β-glucanase / laminarinase; endo-1,3(4)-β-glucanase; licheninase; β-agarase; κ-carrageenase; xyloglucanase; endo-β-1,3-galactanase; [retaining] β-porphyranase; hyaluronidase; endo-β-1,4-galactosidase; chitin β-1,6-glucanosyltransferase; β-transglycosidase; β-glycosidase; β-carrageenase	GH16 (1)	GH16 (1)
Glycoside hydrolase family 18<br>chitinase; lysozyme; endo-β-N-acetylglucosaminidase; peptidoglycan hydrolase with endo-β-N-acetylglucosaminidase specificity; Nod factor hydrolase; xylanase inhibitor; concanavalin B; narbonin	GH18 (3)	GH18 (5)
Glycoside hydrolase family 19<br>Chitinase; lysozyme	GH19 (3)	GH19 (3)

**CAzymes predicted**	***Streptomyces* sp. BT3**	***Streptomyces* sp. BT4**
Glycoside hydrolase family 20<br>β-hexosaminidase; lacto-N-biosidase; β-1,6-N-acetylglucosaminidase; β-6-SO3-N-acetylglucosaminidase	GH20 (2)	GH20 (2)
Glycoside hydrolase family 23<br>(lysozyme type G, peptidoglycan lyase, chitinase)	GH23 (3)	GH23 (3)
Glycoside hydrolase family 25<br>Lysozyme	GH25 (3)	GH25 (3)
Glycosyl hydrolase family 32<br>invertase; endo-inulinase; β-2,6-fructan 6-levanbiohydrolase; endo-levanase; exo-inulinase; fructan β-(2,1)-fructosidase/1-exohydrolase; fructan β-(2,6)-fructosidase/6-exohydrolase; sucrose:sucrose 1-fructosyltransferase; fructan:fructan 1-fructosyltransferase; sucrose:fructan 6-fructosyltransferase; fructan:fructan 6G-fructosyltransferase; levan fructosyltransferase; [retaining] sucrose:sucrose 6-fructosyltransferase (6-SST); cycloinulo-oligosaccharide fructanotransferase	GH32 (1)	GH32 (2)
Glycoside hydrolase family 33<br>sialidase or neuraminidase; trans-sialidase; anhydrosialidase; Kdo hydrolase; 2-keto-3-deoxynononic acid hydrolase / KDNase	GH33 (1)	GH33 (1)
Glycoside hydrolase family 43<br>β-xylosidase; α-L-arabinofuranosidase; xylanase; α-1,2-L-arabinofuranosidase; exo-α-1,5-L-arabinofuranosidase; [inverting] exo-α-1,5-L-arabinanase; β-1,3-xylosidase; [inverting] exo-α-1,5-L-arabinanase; [inverting] endo-α-1,5-L-arabinanase; exo-β-1,3-galactanase; β-D-galactofuranosidase	GH43 (1)	GH43 (1)
Glycoside hydrolase family 46<br>chitosonase	GH46 (1)	GH46 (1)
Glycoside hydrolase family 55	GH55 (1)	GH55 (1)

**CAzymes predicted**	***Streptomyces* sp. BT3**	***Streptomyces* sp. BT4**
exo-β-1,3-glucanase; endo-β-1,3-glucanase		
Glycoside hydrolase family 62<br>α-L-arabinofuranosidase	GH62 (1)	GH62 (0)
Glycoside hydrolase family 63<br>processing α-glucosidase; α-1,3-glucosidase; α-glucosidase; mannosylglycerate α-mannosidase / mannosylglycerate hydrolase; glucosylglycerate hydrolase	GH63 (1)	GH63 (1)
Glycoside hydrolase family 64<br>β-1,3-glucanase	GH64 (1)	GH64 (1)
Glycoside hydrolase family 65<br>α,α-trehalase; maltose phosphorylase; trehalose phosphorylase; kojibiose phosphorylase; trehalose-6-phosphate phosphorylase; nigerose phosphorylase; 3-O-α-glucopyranosyl-L-rhamnose phosphorylase; 2-O-α-glucopyranosylglycerol: phosphate β-glucosyltransferase; α-glucosyl-1,2-β-galactosyl-L-hydroxylysine α-glucosidase; 1,3-Î±-oligoglucan phosphorylase	GH65 (4)	GH65 (3)
Glycoside hydrolase family 77<br>amylomaltase or 4-α-glucanotransferase	GH77 (1)	GH77 (1)
Glycoside hydrolase family 81<br>endo-β-1,3-glucanase	GH81 (1)	GH81 (1)
Glycoside hydrolase family 84<br>(N-acetyl β-glucosaminidase; hyaluronidase; [protein]-3-O-(GlcNAc)-L-Ser/Thr β-N-acetylglucosaminidase	GH84 (2)	GH84 (2)
Glycoside hydrolase family 87<br>mycodextranase; α-1,3-glucanase	GH87 (1)	GH87 (1)
Glycoside hydrolase family 114<br>endo-α-1,4-polygalactosaminidase	GH114 (1)	GH114 (1)
Glycoside hydrolase family 158	GH158 (1)	GH158 (1)

**CAzymes predicted**	***Streptomyces* sp. BT3**	***Streptomyces* sp. BT4**
endo-β-1,3-glucanase		
Glycosyltransferase family 1<br>(UDP-glucuronosyltransferase; zeatin O-β-xylosyltransferase; 2-hydroxyacylsphingosine 1-β-galactosyltransferase; N-acylsphingosine galactosyltransferase; flavonol 3-O-glucosyltransferase; anthocyanidin 3-O-glucosyltransferase; sinapate 1-glucosyltransferase; indole-3-acetate β-glucosyltransferase; flavonol L-rhamnosyltransferase; sterol glucosyltransferase; UDP-Glc: 4-hydroxybenzoate 4-O-β-glucosyltransferase; zeatin O-β-glucosyltransferase; limonoid glucosyltransferase; UDP-GlcA: baicalein 7-O-β-glucuronosyltransferase; UDP-Glc: chalcone 4'-O-β-glucosyltransferase; ecdysteroid UDP-glucosyltransferase; salicylic acid β-glucosyltransferase; anthocyanin 3-O-galactosyltransferase; anthocyanin 5-O-glucosyltransferase; dTDP-β-2-deoxy-L-fucose: α-L-2-deoxyfucosyltransferase; UDP-β-L-rhamnose: α-L-rhamnosyltransferase; zeaxanthin glucosyltransferase; UDP-Glc: flavone-6-C-glucosytransferase; UDP-Glc: cinnamate β-glucosyltransferase; UDP-Glc: hydroxycinnamic acid O-β-glucosyltransferase; UDP-Glc: cinnamoyl O-β-glucosyltransferase; UDP-Arap: flavone-C-arabinosytransferase	GT1 (3)	GT1 (3)
Glycosyltransferase family 2<br>cellulose synthase; chitin synthase; dolichyl-phosphate β-D-mannosyltransferase; dolichyl-phosphate β-glucosyltransferase; N-acetylglucosaminyltransferase; N-acetylgalactosaminyltransferase; hyaluronan synthase; chitin oligosaccharide synthase; β-1,3-glucan synthase; β-1,4-mannan synthase; β-mannosylphosphodecaprenol-mannooligosaccharide α-1,6-mannosyltransferase; UDP-Galf: rhamnopyranosyl-N-acetylglucosaminyl-PP-decaprenol β-1,4/1,5-galactofuranosyltransferase; UDP-Galf: galactofuranosyl-galactofuranosyl-rhamnosyl-N-acetylglucosaminyl-PP-decaprenol β-1,5/1,6-galactofuranosyltransferase; dTDP-L-Rha: N-acetylglucosaminyl-PP-decaprenol α-1,3-L-rhamnosyltransferase; alternating β-1,3/4-N-acetylmannan synthase	GT2 (6)	GT2 (5)

**CAzymes predicted**	***Streptomyces* sp. BT3**	***Streptomyces* sp. BT4**
Glycosyltransferase family 4<br>sucrose synthase; sucrose-phosphate synthase; α-glucosyltransferase; lipopolysaccharide N-acetylglucosaminyltransferase; phosphatidylinositol α-mannosyltransferase; GDP-Man: Man1GlcNAc2-PP-dolichol α-1,3-mannosyltransferase; GDP-Man: Man3GlcNAc2-PP-dolichol/Man4GlcNAc2-PP-dolichol α-1,2-mannosyltransferase; digalactosyldiacylglycerol synthase; 1,2-diacylglycerol 3-glucosyltransferase; diglucosyl diacylglycerol synthase; trehalose phosphorylase; NDP-Glc: α-glucose α-glucosyltransferase / α,α-trehalose synthase; GDP-Man: Man2GlcNAc2-PP-dolichol α-1,6-mannosyltransferase; UDP-GlcNAc: 2-deoxystreptamine α-N-acetylglucosaminyltransferase; UDP-GlcNAc: ribostamycin α-N-acetylglucosaminyltransferase; UDP-Gal α-galactosyltransferase; UDP-Xyl α-xylosyltransferase; UDP-GlcA α-glucuronyltransferase; UDP-Glc α-glucosyltransferase; UDP-GalNAc: GalNAc-PP-Und α-1,3-N-acetylgalactosaminyltransferase; UDP-GalNAc: N,N'-diacetylbacillosaminyl-PP-Und α-1,3-N-acetylgalactosaminyltransferase; ADP-dependent α-maltose-1-phosphate synthase	GT4 (6)	GT4 (6)
Glycosyltransferase family 9<br>lipopolysaccharide N-acetylglucosaminyltransferase; heptosyltransferase	GT9 (1)	GT9 (2)
Glycosyltransferase family 20<br>α,α-trehalose-phosphate synthase [UDP-forming]; Glucosylglycerol-phosphate synthase; trehalose-6-P phosphatase; [retaining] GDP-valeniol: validamine 7-phosphate valeniolyltransferase	GT20 (1)	GT20 (1)
Glycosyltransferase family 28<br>1,2-diacylglycerol 3-β-galactosyltransferase; 1,2-diacylglycerol 3-β-glucosyltransferase; UDP-GlcNAc: Und-PP-MurAc-pentapeptide β-N-acetylglucosaminyltransferase; digalactosyldiacylglycerol synthase	GT28 (2)	GT28 (2)
Glycosyltransferase family 35	GT35 (1)	GT35 (1)

**CAzymes predicted**	***Streptomyces* sp. BT3**	***Streptomyces* sp. BT4**
glycogen or starch phosphorylase		
Glycosyltransferase family 39<br>(Dol-P-Man: protein α-mannosyltransferase)	GT39 (1)	GT39 (1)
Glycosyltransferase family 51<br>Murein polymerase	GT51 (4)	GT51 (3)
Glycosyltransferase family 81<br>NDP-Glc: glucosyl-3-phosphoglycerate synthase; NDP-Man: mannosyl-3-phosphoglycerate synthase; ADP-Glc: glucosyl-2-glycerate synthase; mannosyl-3-phosphoglycerate synthase / [retaining] GDP-Man: 3-phosphoglycerate α-mannosyltransferase	GT81 (1)	GT81 (1)
Glycosyltransferase family 87<br>(polyprenol-P-Man: α-1,2-mannosyltransferase)	GT87 (3)	GT87 (3)
Polysaccharide lyase family 31<br>endo-β-1,4-glucuronan lyase; poly(β-mannuronate) lyase / M-specific alginate lyase	PL31 (1)	PL31 (1)
